# Genome-wide survey of yeast mutations leading to activation of the yeast cell integrity MAPK pathway: Novel insights into diverse MAPK outcomes

**DOI:** 10.1186/1471-2164-12-390

**Published:** 2011-08-02

**Authors:** Patricia Arias, Sonia Díez-Muñiz, Raúl García, César Nombela, José M Rodríguez-Peña, Javier Arroyo

**Affiliations:** 1 Departamento de Microbiología II, Facultad de Farmacia, Universidad Complutense de Madrid, IRYCIS, 28040 Madrid, Spain

## Abstract

**Background:**

The yeast cell wall integrity mitogen-activated protein kinase (CWI-MAPK) pathway is the main regulator of adaptation responses to cell wall stress in yeast. Here, we adopt a genomic approach to shed light on two aspects that are only partially understood, namely, the characterization of the gene functional catalog associated with CWI pathway activation and the extent to which MAPK activation correlates with transcriptional outcomes.

**Results:**

A systematic yeast mutant deletion library was screened for constitutive transcriptional activation of the CWI-related reporter gene *MLP1*. Monitoring phospho-Slt2/Mpk1 levels in the identified mutants revealed sixty-four deletants with high levels of phosphorylation of this MAPK, including mainly genes related to cell wall construction and morphogenesis, signaling, and those with unknown function. Phenotypic analysis of the last group of mutants suggests their involvement in cell wall homeostasis. A good correlation between levels of Slt2 phosphorylation and the magnitude of the transcriptional response was found in most cases. However, the expression of CWI pathway-related genes was enhanced in some mutants in the absence of significant Slt2 phosphorylation, despite the fact that functional MAPK signaling through the pathway was required. CWI pathway activation was associated to increased deposition of chitin in the cell wall - a known survival compensatory mechanism - in about 30% of the mutants identified.

**Conclusion:**

We provide new insights into yeast genes related to the CWI pathway and into how the state of activation of the Slt2 MAPK leads to different outcomes, discovering the versatility of this kind of signaling pathways. These findings potentially have broad implications for understanding the functioning of other eukaryotic MAPKs.

## Background

The capacity to respond properly to external stimuli or environmental conditions is essential for homeostasis in eukaryotes. Signaling pathways, in particular those mediated by mitogen-activated protein kinases (MAPKs), play a key role in these processes. A significant amount of research has been conducted in recent years to characterize these signal transduction pathways, based largely on the model yeast *Saccharomyces cerevisiae *(also known as baker's or budding yeast). Since MAPK pathways are evolutionarily conserved, insights gained from yeast contribute to a better understanding of orthologous pathways in higher organisms. The basic assembly of MAPK pathways is a three-component module conserved from yeast to humans, consisting of three kinases that establish a sequential activation pathway by means of phosphorylation events [1]. The regulation of MAPK signal transduction depends on a variety of mechanisms including scaffold proteins, subcellular localization of different elements and the action of protein phosphatases [2].

In yeast, six MAPK cascades have been identified that mediate the response to different stimuli: (i) pheromones (pheromone response pathway); (ii) nitrogen starvation (filamentous growth pathway); (iii) hyperosmolarity (high osmolarity/glycerol pathway); (iv) *STE *vegetative growth pathway (SVG) [3]; (v) nutrient starvation (spore wall assembly pathway); and (vi) cell wall stress (CWI: cell wall integrity pathway) (see an extensive review of MAPK pathways in [2]). The CWI pathway is essential for maintaining cellular integrity. Therefore, mutations affecting different elements of the pathway lead to cell lysis [4,5]. The maintenance of cellular integrity and morphology, as well as the protection of the cell from adverse environmental conditions, depends on the cell wall, an essential structure that has been characterized extensively in *Saccharomyces cerevisiae *(reviewed in [6,7]). It has three major components: an inner layer of glucans (β-1,3 and β-1,6-glucan), chitin and an outer layer of mannoproteins. These components must be correctly assembled in order to build a fully functional structure [6,8,9].

The essentiality of the cell wall for fungal viability makes it one of the most attractive targets for therapeutic intervention against fungal pathogens [10]. Treatment with cell wall-perturbing agents such as the chitin-binding dyes Congo red and Calcofluor white or zymolyase, which degrades the β-1,3-glucan network, elicits a cellular survival response known as "compensatory mechanism" [11]. This adaptive response includes changes in the yeast transcriptional program that we and other groups have characterized, not only in yeast mutants affected at different stages of cell wall biosynthesis [12], but also in wild-type yeast cells growing under different conditions causing transient cell wall damage [13-18]. The compensatory response leads to, among other effects, an increase in the amount of β-glucan and chitin, the production of several cell wall proteins and changes in the cross-linking between cell wall polymers [19]. Although several signaling pathways contribute to the regulation of cell wall remodeling in order to ensure cell integrity, the regulation of this compensatory response is controlled mainly by the MAPK Slt2p/Mpk1p (hereafter noted as Slt2p) through the cell wall integrity pathway (for a review, see [20,21]). The CWI pathway is regulated through the cell cycle, being also activated in response to a variety of external stimuli and morphological events that cause cell wall stress, such as heat stress, hypo-osmotic shock, mating pheromones, oxidative stress, actin depolymerization, cell wall-related mutations, cell wall-stressing agents, alkaline stress and endoplasmic reticulum (ER) stress [20,22-25].

Several cell membrane proteins (Mid2, Wsc1-4 and Mtl1) [26-28] act as sensors of the CWI pathway. For further intracellular transduction of the activation signal, these sensors interact with the guanine nucleotide exchange factor (GEF) Rom2, activating the small GTPase Rho1, which then activates the yeast protein kinase C (Pkc1). The main role of activated Pkc1 is to trigger a MAPK module. Upon phosphorylation, the MAPKKK Bck1 activates a pair of redundant MAPKKs (Mkk1 and Mkk2), which phosphorylate the MAPK Slt2. Slt2 is a functional homolog of human ERK5 [29], a MAPK that is activated in response to both growth factors and physical and chemical stresses [30,31]. The dually phosphorylated (Thr^190^/Tyr^192^) form of Slt2 activates two transcription factors: the MADS-box transcription factor Rlm1 [32] and SBF, a heterodimeric complex of two proteins, Swi4 and Swi6, which are mainly involved in the regulation of gene expression in G1/S transition [33]. Although Rlm1, activated through phosphorylation by Slt2 [34], is responsible for the transcriptional activation of the majority of the genes induced in CWI adaptation responses, a non-catalytic mechanism of transcriptional activation mediated by SBF through Slt2 has recently been described [35]. This alternative mechanism extends the regulatory roles of the MAPK cascade.

In accordance with the complexity of the cellular processes related to cell wall homeostasis in yeast, cross-talk between distinct MAPK pathways has recently been described. This complicates the simple linear "top-down" concept of signaling pathways. For example, Slt2 is activated in response to hyperosmotic shock through the HOG1 MAPK pathway [36]. Similarly, our group has shown that treating yeast cells with zymolyase also activates Slt2 in a Hog1 pathway-dependent manner [17,37].

Considering that the CWI pathway is activated under cell wall stress, our working hypothesis is that yeast strains lacking genes functionally relevant for cell wall biogenesis or pathway regulation should present a constitutive activation of this route. In this paper, we describe the development of a genomic-wide screening in order to identify genes whose absence produces a functional constitutive activation of the CWI pathway of *Saccharomyces cerevisiae *under vegetative (non-stressed) growth conditions. As a result, we have identified, for the first time at genomic scale, a map of genes that could be functionally related to the CWI pathway. Furthermore, this report gives new insights into the link between the magnitude of MAPK activation and transcriptional induction or cell wall remodeling events. This information can be extended to pathogenic fungi, being useful for future therapeutic purposes.

## Results and discussion

### Large-scale identification of gene deletions that activate the cell wall integrity pathway in yeast

The CWI pathway of *Saccharomyces cerevisiae*, governed by the MAPK Slt2, is triggered under conditions that compromise the integrity of the cell wall. Signaling through this pathway can be monitored by taking advantage of reporters driven by Rlm1-responsive promoters. We have recently developed a transcriptional reporter system, especially suitable for large-scale studies, that potentially allows detecting the functional activation of the CWI pathway [38]. Essentially, this system is based on a plasmid construction (pJS05) that includes a transcriptional fusion of the promoter region of the *MLP1 *gene, one of the main effectors of the transcriptional up-regulation response under cell wall stress, and the coding sequence of the *NAT1 *gene, which encodes resistance to the antibiotic nourseothricin. In other words, the expression of *NAT1 *is controlled by the *MLP1 *promoter; therefore, yeast mutants with a constitutive activation of the CWI pathway transformed with this construction should be able to grow in the presence of higher concentrations of the antibiotic than a wild-type strain.

**Figure 1:**
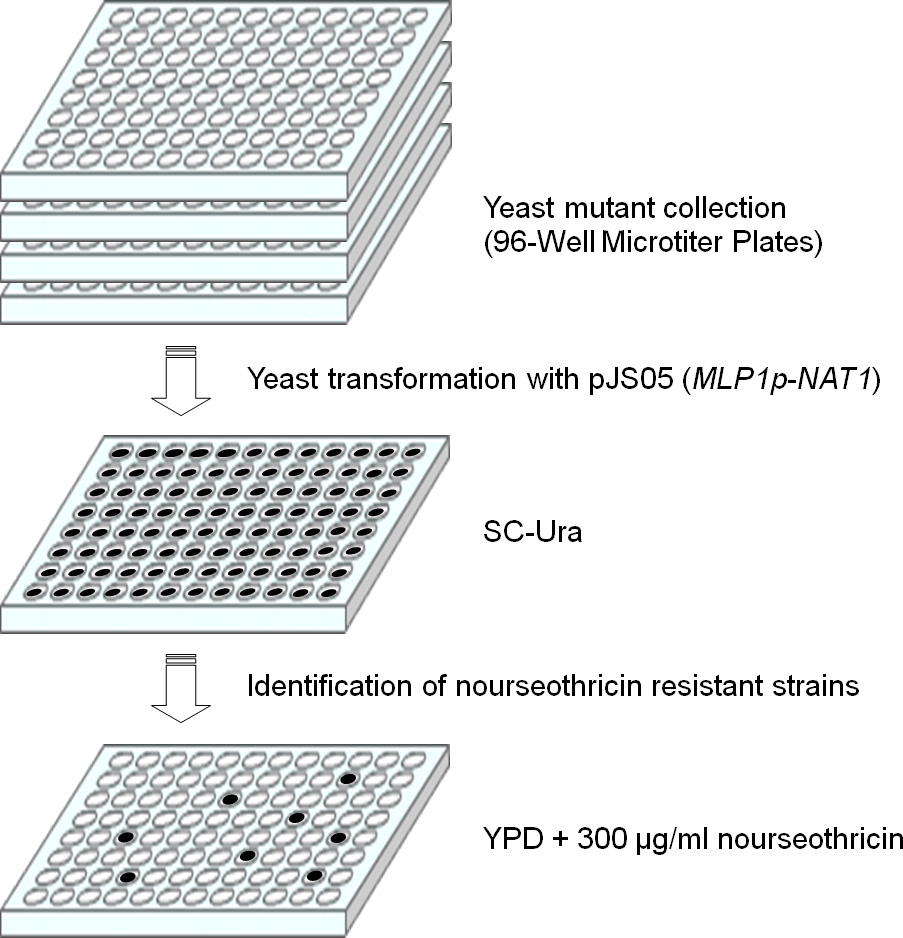
**Overview of the screening strategy designed to identify yeast mutants with constitutive CWI pathway activation**. Yeast mutant strains, transformed with the plasmid pJS05 (*MLP1_P_*-*NAT1*), in which the expression of *MLP1 *is higher compared to the wild-type strain, were identified as resistant to nourseothricin.

Following this approach, we have designed a large-scale screening to identify yeast deletions associated with Slt2-driven transcriptional activation. To achieve this goal, we used the collection of haploid mutant strains in all non-essential genes of *Saccharomyces cerevisiae *(~4800 strains). As represented in Figure 1, we proceeded to transform this collection into a 96-well microplate format with the plasmid containing *MLP1_P_-NAT1*, and the transformed strains were screened for their ability to grow in the presence of 300 μg/ml nourseothricin. This inhibitory concentration of antibiotic was previously determined using selected mutants in which basal levels of Slt2 activation were similar to those found in a wild-type strain (data not shown). After 48-72 hours of growth in a selective medium, 174 mutant strains with hyper-resistance to nourseothricin were identified and selected for further characterization.

### Slt2 phosphorylation levels in nourseothricin-resistant mutants identified in the screening

**Figure 2:**
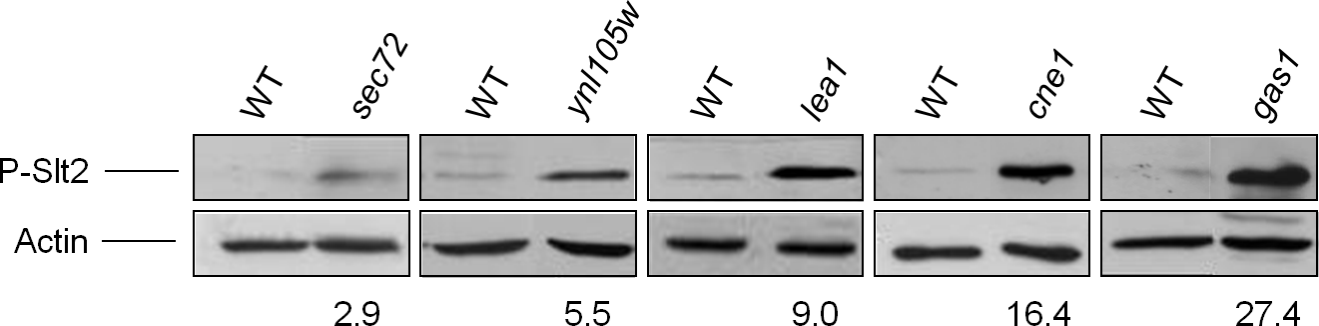
**Representative examples of yeast mutants with increased levels of Slt2 phosphorylation**. Exponential phase cultures of the indicated yeast strains were taken and processed for immunoblotting as described under "Methods". Western blots detecting the phosphorylated form of Slt2 (P-Slt2) and actin as loading control are shown. Numbers correspond to the P-Slt2 fold-change obtained from densitometric quantification of the P-Slt2 bands from Western blots normalized with respect to the actin bands, using the values of the wild-type strain as reference (fold-change set to 1.0).

A reliable method for monitoring signaling through the CWI pathway is to follow the activation state of the MAPK Slt2 by using commercially available antibodies that recognize the dual phosphorylation of conserved threonine and tyrosine residues within the activation loop of Slt2, analogous to Thr^202^/Tyr^204 ^of mammalian p44/p42 MAP Kinase (ERK) [39]. In order to associate the phosphorylation status of Slt2 to *MLP1 *up-regulation, the 174 deletant strains formerly selected were examined following this approach. After densitometric quantification of the phospho-Slt2 bands obtained by Western blotting analysis of the total protein extracts from each mutant strain, 64 mutants recorded higher Phospho-Slt2 levels (at least twofold) than the wild-type strain. The relative amount of phospho-Slt2 in these mutants was distributed over a wide range of values (from twice to thirtyfold) (Table 1). Representative examples of Slt2 activation in different mutants are shown in Figure 2. The complete data set (Western blots) for all the selected mutants is presented in Additional file 1. These data indicate different levels of CWI pathway activation for each mutant, suggesting that yeast cells modulate pathway activation as required by specific stimuli. Remarkably, more than 20% of the mutations identified in a large-scale analysis revealing synthetic lethal interaction with *slt2 *Δ [40] have also been isolated in our screening (Table 1).

**Table 1:** *S. cerevisiae *mutants with increased levels of dually phosphorylated MAP Kinase Slt2

ORF	Gene	P-Slt2	Functional Group	Description
*YAL058W*	*CNE1*	**16.4**	Cell wall and morphogenesis	Calnexin 1. may be involved in a QC process for secretory pathway proteins
*YBL007C*	*SLA1*	**8.1**	Cell wall and morphogenesis	Functions in the assembly of cortical actin cytoskeleton
*YBR078W*	*ECM33*	**14.4**	• Cell wall and morphogenesis	GPI-anchored protein required for CW integrity and mannoprotein biosynthesis
*YCR088W*	*ABP1*	**2.3**	Cell wall and morphogenesis	Actin binding protein that functions in clathrin- and actin-mediated endocytosis
*YDL095W*	*PMT1*	**10.8**	Cell wall and morphogenesis	Mannosyltransferase, first step in O-glycosylation
*YDR349C*	*YPS7*	**16.6**	• Cell wall and morphogenesis	GPI-anchored aspartyl protease involved in maintaining cell wall integrity
*YJL062W*	*LAS21*	**3.6**	Cell wall and morphogenesis	Protein required for addition of a side chain to the GPI core structure
*YJL099W*	*CHS6*	**2.9**	Cell wall and morphogenesis	Protein involved in chitin synthase Chs3p activity
*YJR075W*	*HOC1*	**2.1**	• Cell wall and morphogenesis	Alpha-1,6-mannosyltransferase activity
*YLR319C*	*BUD6*	**2.4**	Cell wall and morphogenesis	Budding 6, required for bipolar budding and involved in bud site selection
*YLR337C*	*VRP1*	**8.6**	Cell wall and morphogenesis	Proline-rich protein verprolin, involved in cytoskeletal organization
*YLR350W*	*ORM2*	**2.6**	Cell wall and morphogenesis	Putative transmembrane protein that may function in the CWI pathway
*YLR370C*	*ARC18*	**5.5**	Cell wall and morphogenesis	Component of the ARP2/3 actin-organizing complex, involved in actin assembly
*YMR307W*	*GAS1*	**27.4**	• Cell wall and morphogenesis	GPI-anchored protein with 1,3-beta-glucanosyltransferase activity
*YNL079C*	*TPM1*	**7.2**	• Cell wall and morphogenesis	Tropomyosin, functions in a variety of processes involving the actin cytoskeleton
*YNL116W*	*DMA2*	**3.4**	Cell wall and morphogenesis	Functions in spindle positioning and septin ring assembly
*YNL192W*	*CHS1*	**2.0**	Cell wall and morphogenesis	Chitin synthase I, has a repair function during cell separation
*YOR002W*	*ALG6*	**6.3**	Cell wall and morphogenesis	Dolichyl-phosphate-mannose-protein mannosyltransferase activity
*YER037W*	*PHM8*	**4.0**	Metabolism	Lysophosphatidic acid phosphatase
*YJL137C*	*GLG2*	**2.3**	Metabolism	Transferase activity, transferring hexosyl groups
*YHL023C*	*RMD11*	**2.9**	Nuclear	Protein possibly involved in meiotic nuclear division
*YPR031W*	*NTO1*	**2.0**	Nuclear	May be involved in chromatin-mediated transcription regulation
*YBR101C*	*FES1*	**2.8**	Protein metabolism	NEF for the cytosolic chaperone Ssa1p. Response to oxidative stress
*YDR069C*	*DOA4*	**2.6**	Protein metabolism	Ubiquitin-specific protease that acts in recycling ubiquitin
*YFL007W*	*BLM10*	**4.0**	Protein metabolism	Proteasome assembly
*YBL024W*	*NCL1*	**5.8**	RNA metabolism	tRNA (cytosine-5-)-methyltransferase activity
*YPL029W*	*SUV3*	**2.0**	RNA metabolism	Mitochondrial RNA helicase of the DEAD box family
*YPL213W*	*LEA1*	**9.0**	RNA metabolism	RNA splicing factor activity
*YDL047W*	*SIT4*	**4.3**	Signal transduction	Serine/threonine phosphatase involved in cell cycle regulation/ion homeostasis
*YDR162C*	*NBP2*	**16.9**	Signal transduction	Negative regulator of the HOG pathway
*YDR389W*	*SAC7*	**7.5**	Signal transduction	GTPase-activating protein for Rho1p
*YER155C*	*BEM2*	**2.4**	• Signal transduction	GTPase-activating (GAP) protein that regulates Rho1p
*YHR082C*	*KSP1*	**2.9**	Signal transduction	Serine/threonine kinase involved in filamentous growth
*YHR206W*	*SKN7*	**2.5**	Signal transduction	Transcription factor involved in the oxidative and osmotic stress responses
*YJR074W*	*MOG1*	**5.4**	Signal transduction	Involved in nuclear protein import, plays a role in osmoregulation via SLN1-SKN7
*YKL126W*	*YPK1*	**2.3**	Signal transduction	Putative S/T protein kinase possibly related with the CWI and sphingolipids
*YLR371W*	*ROM2*	**2.5**	Signal transduction	GDP-GTP exchange factor for Rho1p
*YNL053W*	*MSG5*	**5.4**	Signal transduction	Dual-specificity protein tyrosine phosphatase involved in response to pheromone
*YNR047W*	*FPK1*	**3.9**	Signal transduction	Serine/threonine protein kinase that regulates phospholipid asymmetry
*YLR242C*	*ARV1*	**8.6**	Sphingolipid metabolism	Protein involved in sterol uptake required for normal sphingolipid metabolism
*YER139C*	*RTR1*	**5.2**	Transcription	Protein required for growth at high temperature, RNA polymerase II factor
*YMR136W*	*GAT2*	**10.7**	Transcription	GATA zinc finger transcription factor
*YNL025C*	*SSN8*	**2.2**	Transcription	RNA polymerase II transcription mediator
*YPL042C*	*SSN3*	**3.9**	Transcription	Cyclin-dependent serine/threonine protein kinase of the RNA polymerase II
*YPR065W*	*ROX1*	**4.7**	Transcription	Transcriptional repressor of hypoxic genes
*YPR115W*		**3.6**	Transcription	Phosphoinositide binding. Response to oxidative stress
*YDR200C*	*VPS64*	**3.9**	Transport	Class B vacuolar sorting protein involved in Prc1p trafficking/α-factor secretion
*YFL048C*	*EMP47*	**4.1**	Transport	Golgi and ER membrane protein involved in glycoprotein secretion
*YGR028W*	*MSP1*	**2.1**	Transport	Intra-mitochondrial sorting protein, member of the AAA family of ATPases
*YHR032W*	*ERC1*	**3.5**	Transport	Member of the MatE family. Transporter activity
*YJL133W*	*MRS3*	**5.2**	Transport	Mitochondrial carrier (MCF, family of membrane transporters). Iron transport
*YJR152W*	*DAL5*	**5.7**	Transport	Member of the allantoate family of the major facilitator superfamily (MFS)
*YLR292C*	*SEC72*	**2.9**	Transport	Component of ER protein-translocation subcomplex.
*YCR090C*		**2.0**	Unknown	Unknown function
*YDL173W*		**4.1**	Unknown	Unknown function
*YDR290W*		**4.2**	Unknown	Unknown function
*YGR022C*		**3.9**	Unknown	Unknown function
*YLR250W*	*SSP120*	**5.2**	Unknown	Unknown function
*YLR338W*		**7.2**	Unknown	Unknown function
*YMR119W-A*		**3.2**	Unknown	Unknown function
*YNL058C*		**3.0**	Unknown	Unknown function
*YNL105W*		**5.5**	Unknown	Unknown function
*YNR014W*		**2.5**	Unknown	Unknown function
*YPL158C*		**3.0**	Unknown	Unknown function

Levels of Slt2 phosphorylation (P-Slt2) correspond to the fold-change in each mutant strain relative to the wild-type calculated as described in Methods. Representative data of at least replicated experiments are shown. Functional categories and descriptions were assigned based on the information provided by the BIOBASE Knowledge Library Proteome. Genes were grouped together on the basis of their functional category. Those gene deletions that have been reported to be synthetic lethal with *slt2*Δ are labeled with a black dot.

As shown in Table 1, the functional categorization of mutants identified for constitutive Slt2 activation revealed the most representative functional groups according to the BIOBASE Knowledge Library Proteome and *Saccharomyces *Genome Database classifications, being those involved in cell wall organization and morphogenesis (28%), genes of unknown function (17%), signal transduction (17%), transport (11%) and transcription (9%). The remaining functional categories comprise mutants linked to metabolism (principally of RNA and proteins) and other cellular functions with lower representation in the screening (see Table 1). This distribution is consistent with putative inputs of the pathway, namely, cell wall alterations or regulatory proteins.

**Figure 3:**
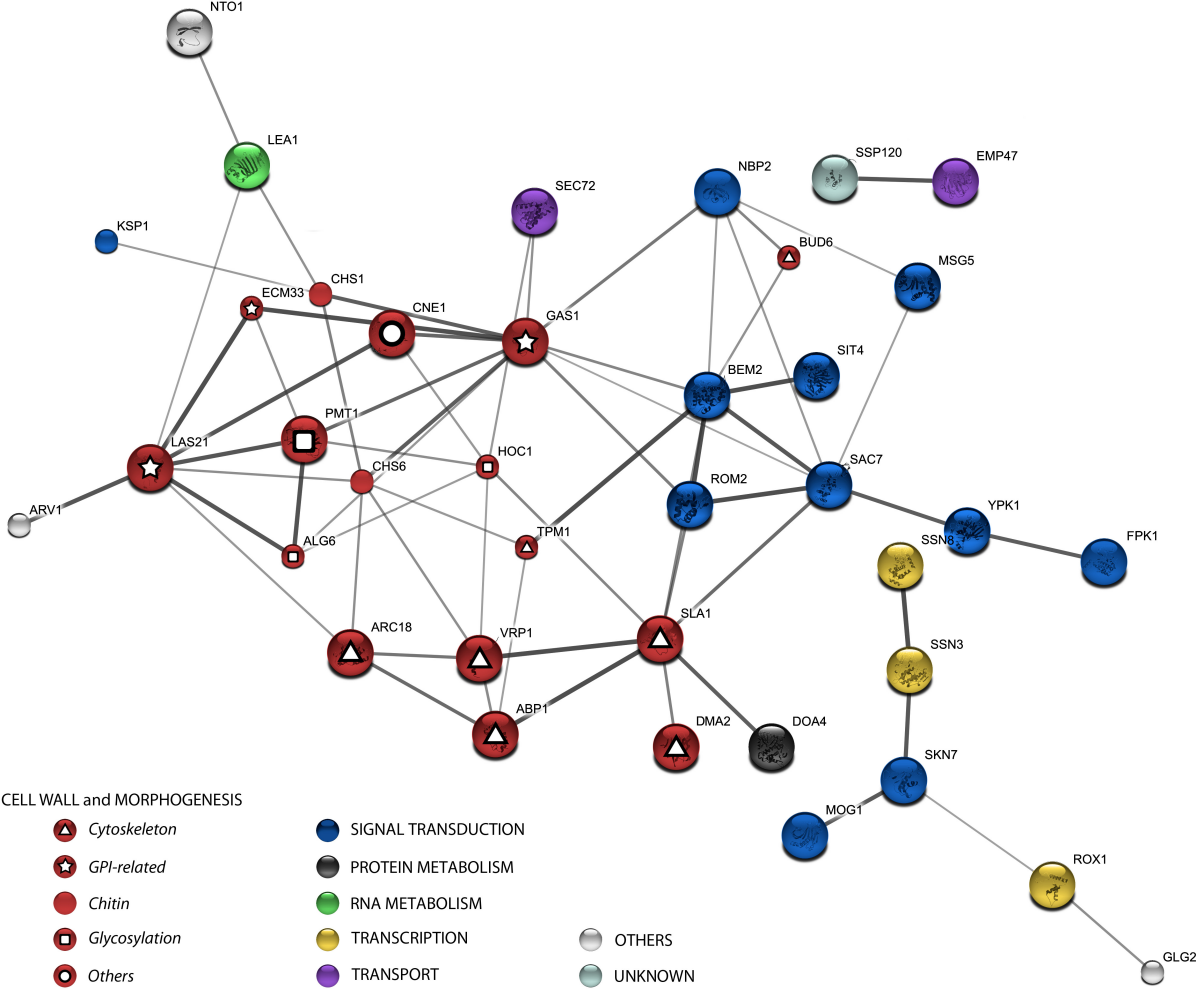
**Interaction network among gene products whose disruption causes Slt2 activation**. Physical and functional interactions were identified computationally using the STRING web resource (8.3 version). Gene products are represented as nodes, shown as filled circles colored according to their functional classification (see Table 1). Interactions are represented as node-connecting edges, shown as lines. Stronger associations are represented by thicker lines. Genes/Proteins with no reported interaction are not shown.

An analysis was conducted of predicted and known interactions between the whole set of genes identified in the screening using the STRING web resource (http://string-db.org) [41]. This tool is very useful for the retrieval of an overall perspective of interacting genes/proteins. As shown in Figure 3, a large number of genes (38 out of 64) showed functional interactions between them. Interestingly, the interaction network was clustered again in the three main functional nodes (cell wall and morphogenesis, signal transduction and transcription) described above. From the large group of 18 mutants related to cell wall and morphogenesis, different subgroups can be highlighted. The first subgroup comprises seven genes (*SLA1*, *ABP1*, *BUD6*, *VRP1*, *ARC18*, *TPM1*, *DMA2*) related to actin cytoskeleton organization. Actin cytoskeleton disruption has been shown to activate the CWI pathway, probably due to the induction of cell wall stress, but the precise molecular mechanism by which Slt2 is stimulated has not been fully established [42]. Moreover, related to this group we found the Doa4 deubiquitinating enzyme (Figure 3), which has been associated with cell morphology and actin cytoskeleton defects. In fact, *DOA4 *had a synthetic genetic interaction with *SLA1 *[43]. A second subgroup encompasses six mutants, including structural cell wall-related proteins. Three of them are glycosylphosphatidylinositol-anchored proteins (GPI-APs) on the cell surface: Gas1, which is a β-1,3-glucanosyltransferase; Ecm33, which is linked to cell wall maintenance; and Yps7, an aspartyl protease. The corresponding deletant strains have severe cell wall defects that may explain their high basal Slt2 activation. In fact, the compensatory response elicited in a *gas1*Δ strain has previously been characterized, and it involves a significant induction in *MLP1 *expression [12]. In agreement with this, *gas1*Δ and *ecm33*Δ mutants have previously been shown to have a constitutively high level of Slt2 phosphorylation [44,45]. Moreover, within this second subgroup we found Las21, an ER membrane protein involved in the synthesis of the GPI core structure. The absence of this protein leads to global cell wall defects. Functionally linked to Las21, the protein Arv1 was identified (Figure 3). This protein is involved in sphingolipid metabolism and has recently been related to the process of GPI synthesis and anchoring [46]. Finally, two proteins related to chitin metabolism, Chs6, which is involved in chitin synthase Chs3 activity, and Chs1 (chitin synthase I), which is required for repairing the chitin septum during cytokinesis [7] were uncovered in our screening. The identification of these mutants is significant since, due to functional redundancy and the existence of gene families, the deletion of certain individual genes encoding cell wall-related proteins does not usually lead to observable phenotypes. A third subset includes *pmt1*Δ, *hoc1*Δ and *alg6*Δ strains. All of them encode mannosyltransferase activities, and their selection in the screening is consistent with the finding that protein glycosylation of cell surface proteins is important for cell wall assembly [7,47]. Interestingly, transcriptional responses to O- and N-glycosylation defects in yeast include the fingerprint of the cell wall damage transcriptional profile [48,49]. In *S. cerevisiae*, mannosyltransferases are highly redundant, being included in different protein families [7,50]. The identification of the abovementioned proteins is indicative of their specific importance in cell wall homeostasis.

Within the group of mutants involved in cellular signaling, we have identified several well-known negative regulators of Slt2 activity, such as the Rho1 GTPase activators Sac7 and Bem2, and protein phosphatases Sit4 and Msg5, which negatively regulate the pathway acting on Pkc1p and Slt2p, respectively [51,52]. We also identified Rom2 in spite of being an activator (Rho1 GEF) of the CWI pathway and the protein kinase Ypk1, a regulator linking sphingolipid signaling and CWI pathway [53]. The effect on Slt2 activation in a *rom2*Δ strain has previously been described [54]. These authors hypothesized that *rom2*Δ mutants have a defective cell wall due to a decreased activity of the CWI pathway, and this alteration may triggers Slt2 activation through Rom2-independent mechanisms. The singling out of these mutants further validates our screening for discovering novel potential regulators of the CWI pathway. This is the case of Nbp2, Ksp1, Fpk1, Mog1 and Skn7. Nbp2 acts as a negative regulator of the HOG pathway by recruiting Ptc1 phosphatase to Hog1 [55], and has been involved in cortical ER inheritance via Slt2 [56], the protein kinase Ksp1 has recently been linked to filamentous growth in haploid yeast cells [57], and Fpk1 (flippase kinase 1) regulates phospholipid membrane translocation [58]. Skn7 is a multifunctional transcription factor, as reflected by its ability to partner a variety of other transcriptional regulators under different conditions. It has previously been shown that Skn7 may be activated by Rho1p in response to cell wall stress [59], whereas Mog1p is a protein involved in nucleocytoplasmic transport. At the same time, Mog1 is required for optimal recruitment of Skn7 to specific gene promoters [60]. The fact that Slt2 is hyperactivated in these mutants suggests novel connections between the CWI pathway and the cellular processes controlled by these elements.

Regarding the set of mutants related to transcription, many of the proteins identified are involved in RNA polymerase II dependent transcription controlling responses to a variety of conditions such as, heat stress (*RTR1*), oxidative stress (*YPR115w*) and anaerobic conditions (*ROX1*). Additionally, as visualized in the network map (Figure 3), there is a connection between the transcription factors Skn7 and Rox1, with both participating in the transcriptional response to oxidative stress [61]. Also, we identified two components of a module of the mediator complex (Ssn3 and Ssn8), involved in the regulation of Skn7 activity [62]. The appearance of these mutants in our study suggests a functional link between them and the CWI pathway. These insights enable an association to be made between this MAPK pathway and additional stressful cellular events. In this regard, it is also worth to mention that some of the mutants identified in our screening have previously shown altered sensitivity to osmotic stress (*phm8*Δ, *glg2*Δ and *mrs3*Δ) or heat stress (*ncl1*Δ, *suv3*Δ and *vps64*Δ).

A connection has been described between the CWI pathway and endoplasmic reticulum (ER) stress. When a cell encounters conditions that increase misfolded proteins, the Unfolded Protein Response (UPR) is activated to compensate for high levels of ER stress [63]. The Slt2 MAPK pathway is activated during ER stress [23], while UPR is activated by signaling through the CWI pathway during cell wall stress [64]. Moreover, a second pathway, the ER stress surveillance pathway (ERSU) independent of the UPR, has recently been linked to Slt2 activation [65]. According to our results, in some of the selected mutants these mechanisms of Slt2 activation could be involved. In fact, Emp47 is required for the export of specific glycoprotein cargo from the endoplasmic reticulum, Sec72 is involved in targeting secretory proteins to ER, and Orm2 is a protein related to lipid homeostasis and protein quality control, being required for resistance to agents that induce UPR [66]. In addition, Orm2 interacts with Slt2, but the biological significance of this interaction is still unknown [67]. Finally, we found the *cne1 *mutant to be associated with ER quality control mechanisms. Cne1 is a calnexin homologue of *Saccharomyces cerevisiae *that may play a part in the degradation of misfolded glycoproteins.

The identification of deletant strains in genes whose function remains uncharacterized and those not previously associated to cell wall integrity, both recording an increase in Slt2 phosphorylation, was of special interest since they could be putatively associated with cell wall construction or regulation. To further investigate this possibility, a phenotypical analysis was conducted on the 11 deletant strains corresponding to genes of unknown function and 15 without clear cell wall phenotypes reported in yeast databases. Thus, the sensitivity to Congo red, caspofungin, hygromycin B, caffeine and SDS was determined. These compounds affect cell integrity through different modes of action, whereby the dye Congo red interferes with proper cell wall assembly [68], caspofungin consists of a β-1,3-glucan synthase inhibitor, hygromycin B hypersensitivity has been associated with glycosylation defects [69], SDS is a detergent that affects membrane stability and also, indirectly, cell wall construction (increased accessibility) [70], and caffeine is a substance that indirectly activates the CWI pathway in a TOR1-dependent fashion [71]. Eventually, 15 out of 26 strains analyzed displayed altered sensitivity in at least one of the tests described (Table 2), suggesting that the activation of the CWI pathway in these mutants could be due to direct or indirect cell wall alterations. Interestingly, these mutants generally had more than one phenotype supporting the existence of relevant cell wall damage. In contrast, identification of mutants without apparent cell wall defects could be related to the possibility of CWI pathway activation by other stimuli. In this regard, the coordination under specific growth conditions between the CWI and other regulatory MAPK pathways has been extensively reported [20,25]. Further supporting this, Harrison and colleagues [42] suggested that the activation of the CWI pathway by different stresses, rather than operating in a linear "top-down" manner, would provide lateral inputs that impact this regulatory pathway at different levels. Moreover, recent findings connect Slt2 MAPK to DNA damage responses [72].

**Table 2:** Sensitivity test on yeast deletant strains

	Sensitivity				
	
ORF/Gene	Congo red	Caspofungin	SDS	Caffeine	Hygromycin B
*YCR090C*	---	---	---	---	---
*YDL173W*	---	---	**S**	**SS**	---
*YDR290W*	**SSSS**	**SSS**	**S**	---	**SS**
*YGR022C*	---	**SSS**	**S**	---	---
*YLR250W*	---	---	---	---	---
*YLR338W*	**SSSS**	**R**	**SS**	**SSSSS**	**SSSSS**
*YMR119W-A*	---	---	---	---	---
*YNL058C*	---	---	---	---	---
*YNL105W*	---	---	---	---	---
*YNR014W*	---	---	---	**R**	---
*YPL158C*	---	**SSS**	---	**SS**	---

*ERC1*	---	---	---	---	---
*FES1*	**SSSSS**	---	**SSSSS**	**SSSSS**	---
*FPK1*	---	---	---	**R**	---
*GAT2*	**SS**	---	**S**	---	**SSSSS**
*GLG2*	---	---	---	---	---
*KSP1*	---	---	---	---	---
*LEA1*	**SS**	---	---	**SSSS**	**SSS**
*MOG1*	---	**SS**	**SS**	**SS**	**SSSSS**
*MSP1*	---	---	---	---	---
*PHM8*	---	---	---	---	**SSS**
*RMD11*	---	---	**S**	---	---
*ROX1*	---	---	---	---	**SSSS**
*SEC72*	---	---	---	---	---
*SUV3*	---	---	---	---	**SSS**
*YPR115W*	---	---	---	---	---

Sensitivity to Congo red (100 μg/ml), Caspofungin (40 ng/ml), SDS (200 μg/ml), Caffeine (12 mM) and Hygromycin B (50 μg/ml) using a spot dilution assay (see Methods for details) is shown. Hypersensitivity levels are shown as the number of spot dilutions ("S") in which no cell growth is scored. "R" refers to a resistant phenotype and "---" indicates no difference in growth relative to the wild-type strain. Genes of unknown function are grouped in the upper part of the table.

### Differences in chitin content between mutants with Slt2 activation

**Figure 4:**
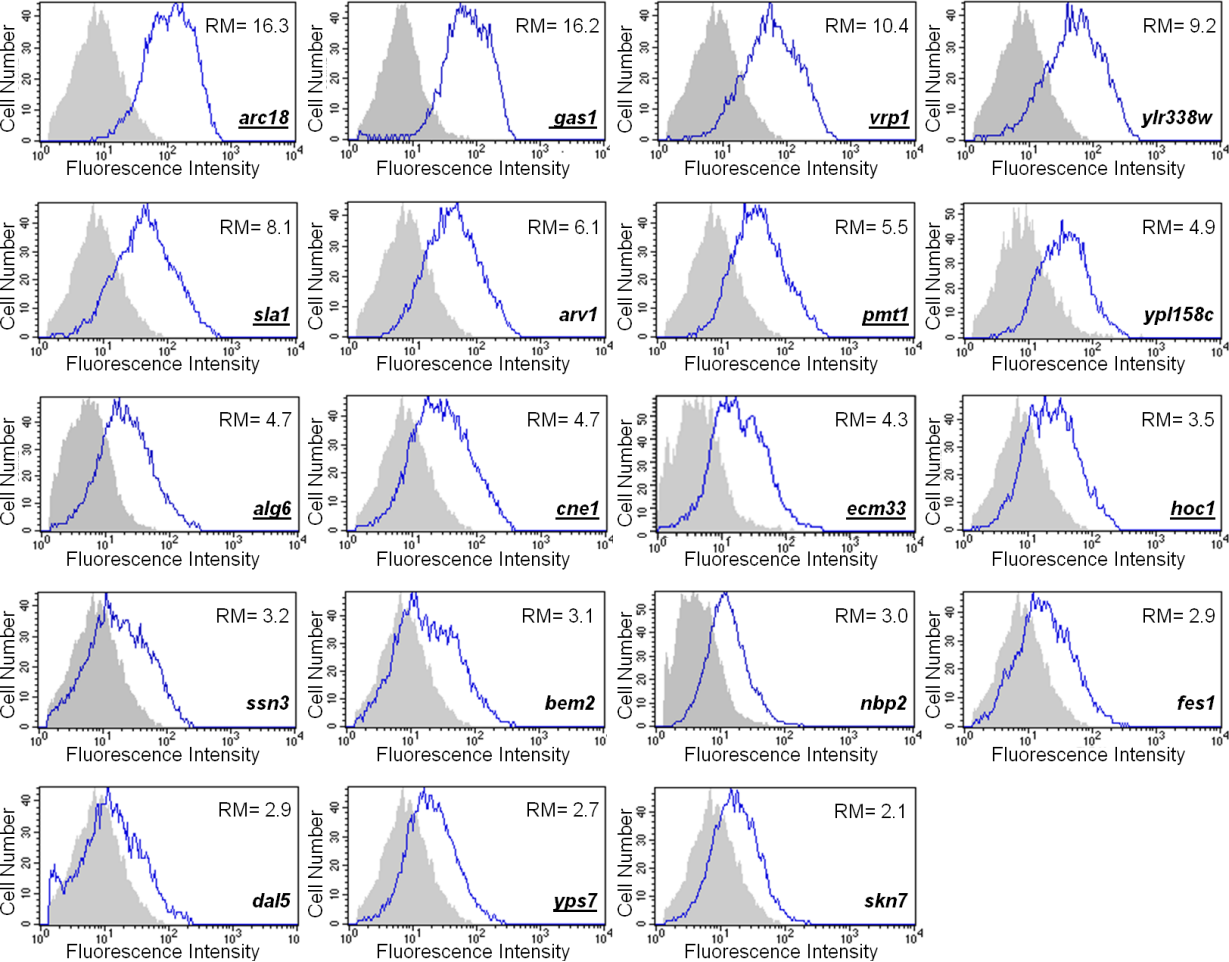
**Identification of mutants with increased chitin content**. Depicted histograms correspond to flow cytometry analysis of yeast cells after treatment with the chitin-binding fluorescent dye, Calcofluor white (5 μg/ml for 10 min). Representative experiments of mutants are shown in which chitin content was found to be higher compared to the wild-type strain. For each mutant (blue line), a histogram corresponding to the wild-type strain (filled area) handled in parallel is included in each representation. The name of genes functionally related to cell wall integrity and morphogenesis is underlined. "RM" refers to the ratio of means of fluorescence intensity for each cell population (mutant/wt).

The yeast cell wall normally contains approximately 2% chitin. However, certain mutations affecting cell wall stability increase chitin levels to as much as 20% of total wall polymers [73]. As this emergency response for cell wall repair is dependent on CWI pathway signaling, it prompted us to assess this response in the whole group of 64 mutants with basal activation of the pathway. Chitin content was measured by means of flow cytometry after staining the cells with the chitin-binding dye, Calcofluor white (CW). This is a reliable and sensitive method for chitin determination, since it has been established a linear relationship between fluorescence from yeast cells stained with CW measured by flow cytometry and the biochemical determination of chitin [74]. Remarkably, 19 (~30%) strains contained more than twice the amount of chitin than the wild-type strain (Figure 4), denoting that a cell wall compensatory mechanism is triggered in these cells. As described above for Slt2 activation, a wide range of chitin content was observed, suggesting that increased deposition of this polymer is adapted to specific cellular requirements. In some of these mutants (*nbp2*Δ, *sla1*Δ, *vrp1*Δ, *ylr338w*Δ, *gas1*Δ and *arc18*Δ) elevated chitin levels and genetic interactions with mutations involved in chitin synthesis have previously been described [75]. In contrast, the remaining 45 mutants did not record an evident increase in CW binding. Interestingly, about half of those mutants with increased chitin levels have been related functionally to cell wall and/or morphogenesis, whereas within the group of mutants lacking significantly increased chitin deposition only 18% were assigned to this functional group. These data suggest a functional link between the chitin-related mechanism and activation of the CWI pathway by cell wall and morphogenesis alterations. Eight out of ten mutants with maximum Slt2 phosphorylation, most of them cell wall related (see Table 1), recorded higher chitin levels, reinforcing the idea that this polymer plays a key role in yeast for salvaging the cell under conditions that jeopardize cell integrity.

**Figure 5:**
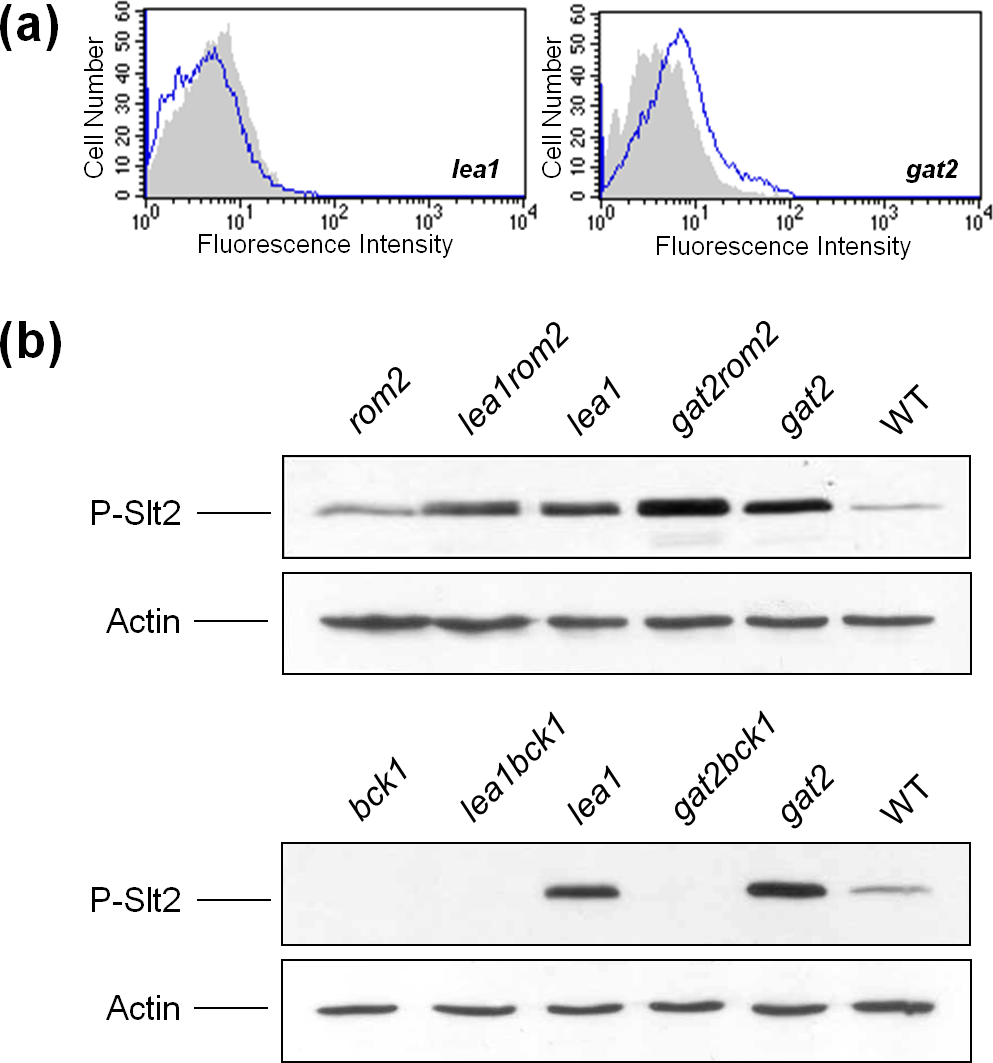
**Slt2 activation in *gat2*Δ and *lea1*Δ mutants is not transmitted via Rom2 nor related to variations in chitin content**. **(a) **Analysis of chitin content of *gat2*Δ and *lea1*Δ cells after Calcofluor white staining by means of flow cytometry as described in Figure 4. **(b) **Western blots detecting Phospho-Slt2 (P-Slt2) and actin as loading control in total protein extracts from single (*gat2*Δ and *lea1*Δ) and double (*lea1*Δ*rom*2Δ, *gat2*Δ*rom2*Δ, *lea1*Δ*bck1*Δ and *gat2*Δ*bck*1Δ) mutant strains.

However, the identification of mutants with no significant variations in the amount of chitin indicates that CWI pathway activation could be due to alternative stimuli or internal inputs on this signaling route. In this regard, *gat2*Δ and *lea1*Δ mutants are two examples of special interest because they have strong Slt2 activation without affecting their chitin content (Table 1 and Figure 5a), despite they showed cell wall alterations. *GAT2 *encodes for a poorly characterized putative zinc finger transcription factor, while *LEA1 *gene product is involved in RNA splicing, although its null mutation shows synthetic sick interaction with several cell wall related genes, such as *CHS1 *or *CHS5 *[40]. In order to gain further insights into the origin of pathway activation in these mutants, we decided to construct double mutants deleting *ROM2 *or *BCK1 *in the *gat2*Δ and *lea1*Δ backgrounds. Rom2 is the major GEF for Rho1 that is responsible for relaying signals from cell surface to Rho1 for its activation [76,77], while Bck1 is the first element of the CWI pathway MAPK module. These mutants allowed us to distinguish whether the phosphorylation of Slt2 was the result of cell wall stress sensing or otherwise took place directly through the MAPK module of the route independently of upstream elements. After investigating Slt2 phosphorylation in single (*gat2*Δ and *lea1*Δ) and double (*gat2*Δ*rom2*Δ; *gat2*Δ*bck1*Δ; *lea1*Δ*rom2*Δ; *lea1*Δ*bck1*Δ) mutants, it was evident that MAPK activation was fully dependent on Bck1, whereas the lack of Rom2 did not affect the Slt2 activation in *gat2*Δ and *lea1*Δ strains (Figure 5b). Nevertheless, participation of other Rho1 GEFs like Rom1 or Tus1 can not be ruled out. This is in contrast to the activation of the CWI pathway by the cell wall stress caused by Congo red, in which Rom2 is demanded for Slt2 activation [37]. These results support the notion that particular cell wall alterations could trigger specific adaptive responses through the CWI pathway.

### MAPK phosphorylation vs. transcriptional activation

**Figure 6:**
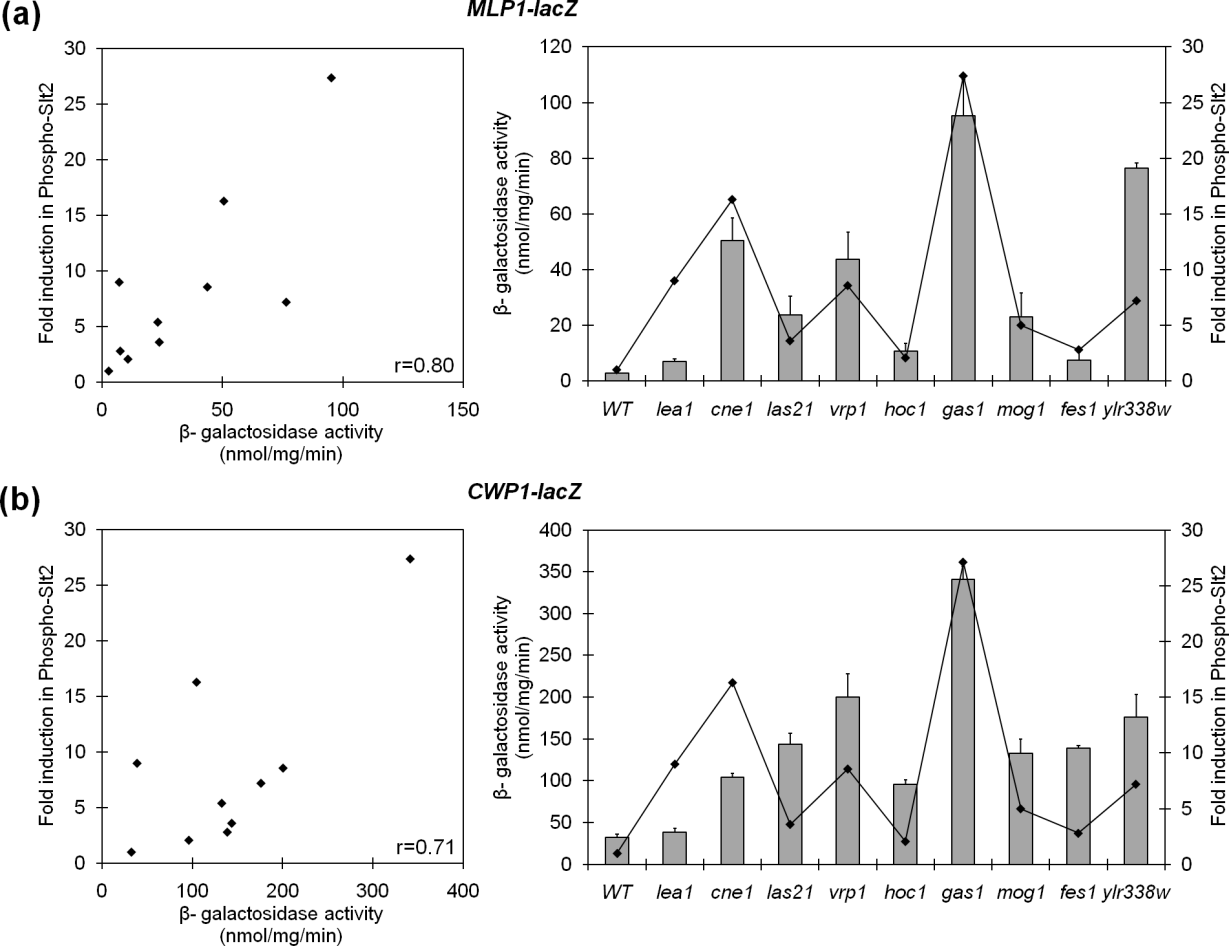
**Examination of transcriptional activation and Slt2 phosphorylation in mutants selected from the screening**. Bars correspond to levels of *MLP1-lacZ ***(a) **and *CWP1-lacZ ***(b) **expression observed in the indicated deletion mutants and the wild-type strain. Three independent experiments were carried out to calculate the means of β-galactosidase activity and standard deviations. The line graph represents the fold-change in Slt2 phosphorylation for each mutant relative to the values observed in the wild-type strain (1x) as described in Table 1. 2D scatter plots of fold-change in phospho-Slt2 vs transcriptional activation are shown on the left side, including the Pearson's correlation coefficient (r).

An important aspect we wanted to address about the functioning of the CWI pathway was the association between the magnitude of Slt2 phosphorylation and the concomitant effect on gene expression. To achieve this goal, a selected group of mutants, representing different levels of Slt2 activation, were transformed with a reporter construction where the promoter region of the *MLP1 *gene was fused to the *lacZ *coding sequence (*MLP1_P_*-*lacZ*) and transcriptional activation was studied under standard growth conditions by measuring β-Galactosidase activity. As shown in Figure 6a, a good correlation between the expression levels of *MLP1 *and Slt2 phosphorylation (Pearson's correlation coefficient of 0.8) was observed except for the mutant *lea1*Δ, in which the reporter expression was significantly lower than expected from the MAPK phosphorylation status. Similar results were obtained when using the *CWP1 *promoter, another reporter of the CWI pathway (Figure 6b). In this regard, comparable behavior was recently described for the mutant *msg5*Δ in which Slt2 phosphorylation is not associated with Rlm1-dependent transcription [78]. On the basis of this observation, the existence of additional *S. cerevisiae *mutants with the same behavior cannot be ruled out.

In contrast to the group of mutants described above with increased levels of phospho-Slt2, another group of 110 mutants selected in the screening did not have detectable differences in Slt2 phosphorylation with respect to the wild-type strain. In order to confirm the nourseothricin resistance of these strains, we transformed them all with the reporter construct (*MLP1_P_-NAT1*) and carried out minimal inhibitory concentration (MIC) assays using a microdilution method. Eventually, 38 mutant strains recorded higher antibiotic MIC values than that of the wild-type (Additional file 2), confirming the phenotype of nourseothricin resistance, whereas all the other mutants behaved the same as the wild-type strain. Bearing in mind that this phenotype was also confirmed for the 64 mutants with increased phosphorylation of Slt2, this group probably includes mutants with antibiotic resistance by non-CWI-related mechanisms, such as alternative effects on *MLP1 *expression or intrinsic antibiotic resistance.

**Figure 7:**
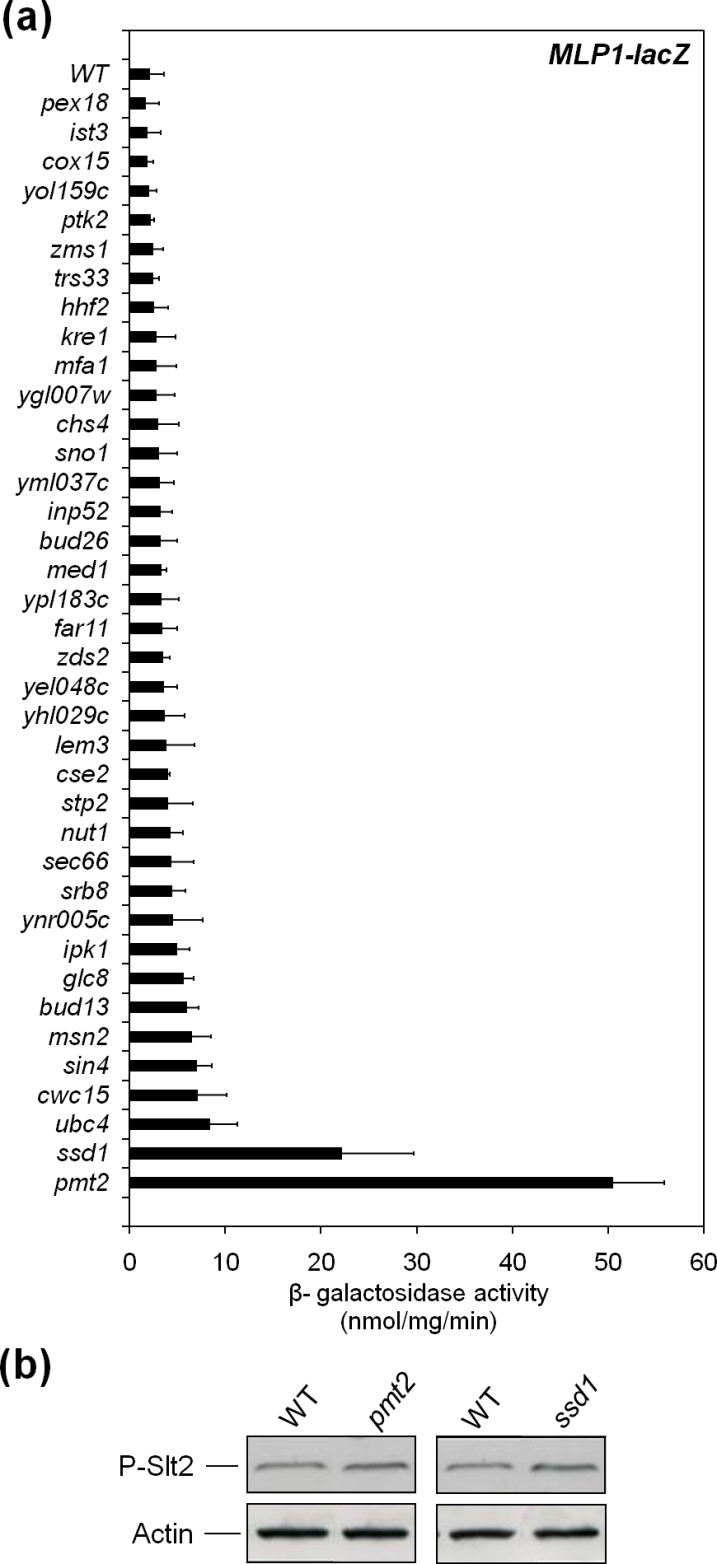
**Examination of *MLP1 *expression in mutants lacking Slt2 activation**. **(a) **Expression of *MLP1-lacZ *was determined in wild-type and the indicated deletant strains growing to mid-log phase in a rich medium. Each value represents the mean and standard deviation of three independent transformants. **(b) **Slt2 phosphorylation was examined by immunoblotting total extracts from exponential cultures of the wild-type strain and *pmt2*Δ and *ssd1*Δ mutants growing in YEPD with an anti-phospho-p44/p42 MAPK antibody. The protein load was monitored using a mouse anti-actin mAb.

**Figure 8:**
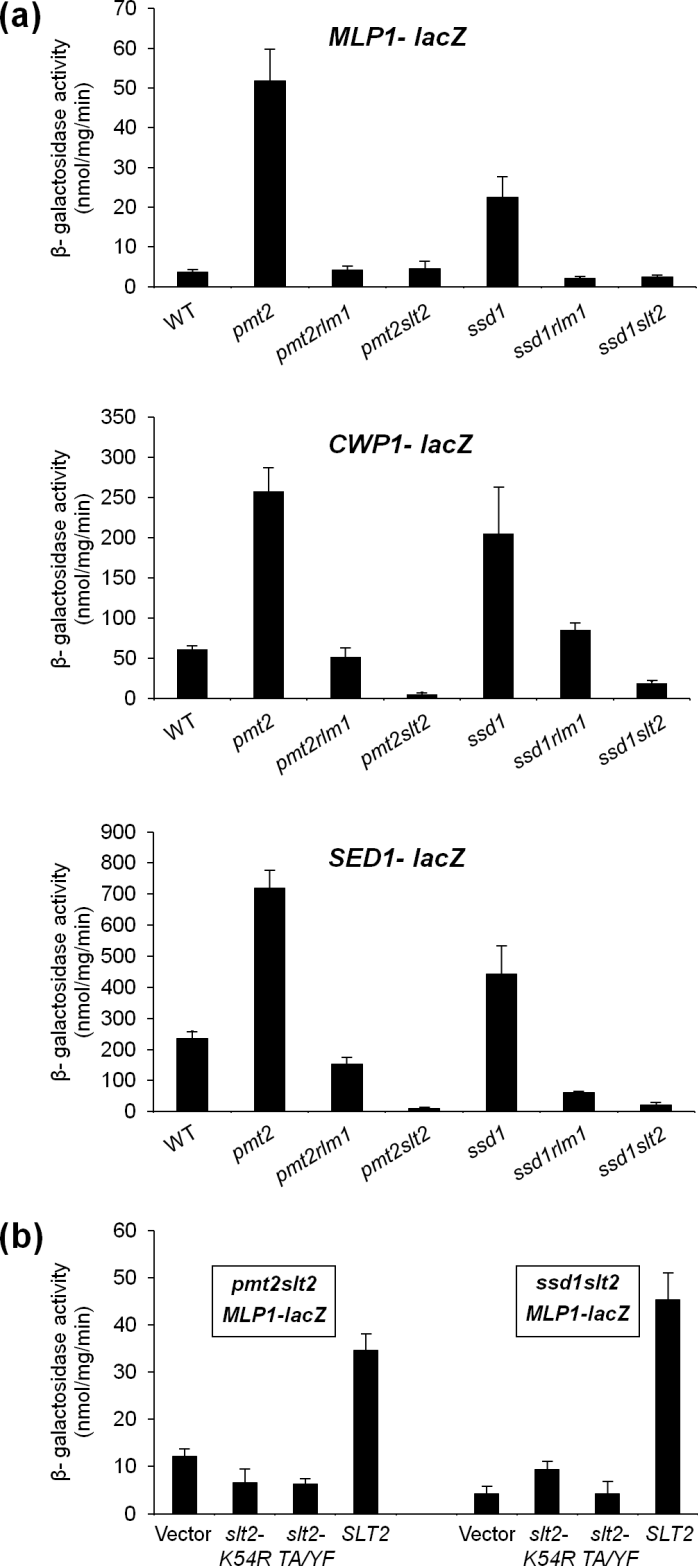
**Transcriptional response observed in mutants *pmt2*Δ and *ssd1*Δ is dependent on Slt2 activity**. **(a) **Expression of *MLP1-lacZ, CWP1-lacZ *and *SED1-lacZ *was determined in wild-type strain and *pmt2*Δ, *pmt2*Δ*rlm1*Δ, *pmt2*Δ*slt2*Δ, *ssd1*Δ, *ssd1*Δ*rlm1*Δ and *ssd1*Δ*slt2*Δ mutant cells growing in a rich medium under standard conditions. **(b) **The *MLP1-lacZ *reporter plasmid was co-transformed with centromeric plasmids bearing the wild-type *SLT2 *allele, inactive mutant alleles of *SLT2 *(*slt2/mpk1-K54R *and *slt2/mpk1-TA/YF*) or the empty vector (pRS315) into *pmt2*Δ*slt2*Δ and *ssd1*Δ*slt2*Δ strains, and β-galactosidase activity was measured in crude extracts. Each value represents the mean and standard deviation of three independent transformants.

To further investigate the molecular mechanism involved in the group of antibiotic resistant strains without detectable variation in phospho-Slt2 levels, the *MLP1_P_-lacZ *reporter was used to monitor levels of expression of *MLP1 *in this set of mutants. For the majority of the mutants, *MLP1 *expression levels were low and in general higher than the wild-type strain (Figure 7a). However, mutant strains *ssd1*Δ and *pmt2*Δ had very high levels of *MLP1 *expression (Figure 7a) in spite of very slight, if any, Slt2 activation (Figure 7b). Pmt2 catalyzes the first step in O-mannosylation of target proteins [79] and *SSD1 *has been linked to cell wall integrity [80,81]. To elucidate a possible participation of the CWI pathway in the activation of the gene expression in these mutants, we proceeded to generate *pmt2*Δ*slt2*Δ, *pmt2*Δ*rlm1*Δ, *ssd1*Δ*slt2*Δ and *ssd1*Δ*rlm1*Δ double mutants. By using these strains transformed with plasmids containing transcriptional fusions of *MLP1*, *CWP1 *and *SED1 *to *lacZ*, we were able to delimit the requirement of the CWI MAPK and its main transcription factor (Rlm1) for the observed transcriptional up-regulation. As shown in Figure 8a, gene activation in the absence of both CWI pathway elements, Slt2 or Rlm1, was completely annulled compared to *pmt2*Δ and *ssd1*Δ single mutants. These results point to the existence of situations where undetectable changes in MAPK activation (Figure 7b) give rise to remarkable consequences at gene expression levels. As further proof of the essentiality of Slt2 activity in the transcriptional response observed in the *pmt2*Δ and *ssd1*Δ strains, we took advantage of two mutant forms of MAPK Slt2. The first was a variant K54R, consisting of a mutation within the ATP-binding site, which blocks the catalytic activity of the protein. The second one (TA/YF) eliminates the phosphorylation of Slt2 by upstream MAPKKs Mkk1/Mkk2. Both alleles, borne on centromeric plasmids, were unable to restore *MLP1_P_*-*lacZ *expression in *pmt2*Δ *slt2*Δ and *ssd1*Δ *slt2*Δ strains (Figure 8b), indicating that signaling through active Slt2 was imperative. These results sustain a mechanism of MAPK signaling in which high levels of transcriptional induction through Rlm1 are not necessarily associated with MAPK phosphorylation levels. This is relevant for CWI pathway-related studies since the phosphorylation status of Slt2 might not always reflect the real pathway outcomes. Further studies will be necessary to characterize the mechanism involved.

## Conclusions

The fine and specific tuning of transduction pathways to ensure yeast cell survival under adverse environmental conditions is essential. Our study contributes significantly to a better understanding of how yeast cell responses to those conditions that jeopardize cell wall integrity or alter its regulation through the CWI pathway. This work has allowed us to identify, at genomic scale, a cluster of genes whose absence induces the transcriptional activation associated with the cell wall integrity compensatory mechanism. Increased levels of phosphorylated MAPK Slt2 were found in a large group of these mutants, in agreement with their direct or indirect association with cell integrity. In fact, the main cluster of genes within this group is related to cell wall biogenesis, morphogenesis and signal transduction. Of special interest are those genes detected by the screening that have not previously been involved in cell wall integrity, particularly those of unknown function, as well as genes related to transcription, RNA and protein metabolism and transport.

Although an increase in chitin content has been described as one of the effector mechanisms within the compensatory response, our results show that there is not always a correlation between activation of the CWI pathway and chitin levels. This effect is probably dependent on the stimuli involved. In fact, within the group of mutants with higher chitin levels, the functional group of genes related to cell wall and morphogenesis is overrepresented, suggesting a functional link between the activation of the chitin deposition-mediated mechanism and cell wall defects.

Another aspect of interest is the lack of uniformity of the magnitude of MAPK activation and the transcriptional outputs in the mutants analyzed, suggesting that their modulation could be relevant for cellular adaptation to specific circumstances. Although levels of MAPK activation generally correlate with transcriptional up-regulation, there are also, and unexpectedly, mutants with a transcriptional activation dependent on a functional Slt2 MAPK and the transcription factor Rlm1, despite not having significant levels of phospho-Slt2. Bearing in mind that this phenomenon could take place in other circumstances, MAPK pathway related studies should seek information on both MAPK activation and gene expression to discover whether the pathway plays a role under specific conditions.

## Methods

### Strains and media

Experiments were performed with the full collection of *Saccharomyces cerevisiae *strains (BY4741 background, *MAT***a**; *his3*Δ*1*; *leu2*Δ*0*; *met15*Δ*0*; *ura3*Δ*0*) individually deleted in all of the ORFs identified in this organism (4840) that were replaced by the Geneticin resistance-codifying *Kan*MX4 module. This collection was provided by Euroscarf (Germany). Double mutants *slt2*Δ*pmt2*Δ, *slt2*Δ*ssd1*Δ, *rlm1*Δ*pmt2*Δ, *rlm1*Δ*ssd1*Δ, *gat2*Δ*rom2*Δ, *lea1*Δ*rom2*Δ, *gat2*Δ*bck1*Δ and *lea1*Δ*bck1*Δ were primarily obtained by directly replacing the genes *SLT2 *or *RLM1 and ROM2 or BCK1 *in the corresponding *pmt2*Δ or *ssd1*Δ and *gat2*Δ or *lea1*Δ single-deletant-strains, respectively, with the *HIS3 *marker using the SFH PCR-based method described by Wach and colleagues [82]. Alternatively, *slt2*Δ::*HIS3, rlm1*Δ::*HIS3*, *rom2*Δ::*HIS3 *or *bck1*Δ::*HIS3 *strains constructed in the wild-type BY4742 background (*MAT*α) were crossed with the single deletants of interest to generate the corresponding heterozygous disruptants. After sporulation and tetrad analyses of these strains with standard yeast genetic techniques, haploid double-mutant segregants were selected. Correct ORF replacements were verified by PCR.

For routine cultures, *S. cerevisiae *was grown on YEPD (2% glucose, 2% peptone, 1% yeast extract) or SC-Ura/Leu medium (0.17% yeast nitrogen base, 0.5% ammonium sulfate, 2% glucose and uracil/leucine drop**-**out mix).

The *Escherichia coli *strain used as plasmid host was DH5α. For selective growth, bacteria were grown on LB medium containing 100 mg/l ampicillin.

Standard procedures were used for yeast genetic and DNA manipulations.

### DNA manipulation and plasmids

General DNA manipulation methods were performed using standard techniques [83]. Whenever necessary, sequence verification of plasmid constructions was carried out on an automated 3730 DNA Analyzer (Applied Biosystems). The plasmid pJS05 [38] contains the promoter region of the gene *MLP1 *(*YKL161c*) fused to the *NAT1 *ORF (encoding for resistance to the antibiotic nourseothricin). Plasmids *MLP1_P_*-*lacZ*, *CWP1_P_*-*lacZ *and *SED1_P_*-*lacZ *including the promoter region of *MLP1*, *CWP1 *and *SED1 *genes fused to *lacZ *have been described previously [17]. Plasmids bearing the Slt2/Mpk1 alleles TA/YF (p2190), K54R (p2193) and the wild-type gene (p2188) were previously described [35].

### Screening for yeast mutants displaying increased basal *MLP1 *expression

Initially, the full collection of yeast mutant strains distributed in seventy-five 96-well microtiter plates (Nunclon) were transformed with the plasmid pJS05 following a protocol of microtiter plate transformation [84], using SC-Ura as selection medium. A new set of plates containing 100 μl of fresh SC-Ura medium were then inoculated with 5 μl of the original transformation cultures and allowed to grow at 28°C for 24 h to be used to inoculate the plates for the screening for *MLP1 *induction. Thus, 5 μl of these pre-inoculums were used to inoculate the definitive set of microtiter plates containing 95 μl of YEPD plus 300 μg/ml of nourseothricin (Werner BioAgents, Germany) per well. The plates were incubated in a static culture at 28°C for 48-72 h and growth was determined by measuring absorbance at 550 nm in each well with a microplate reader (Model 680, Bio-Rad). The parameters described above, including incubation times, inoculum size and antibiotic concentration, were previously optimized in order to prevent the growth of the wild-type strain (unaltered *MLP1 *expression).

### Nourseothricin susceptibility testing

Selected yeast mutants and the wild-type strain, as control, were individually transformed with the plasmid pJS05 following standard yeast genetic methods. Antibiotic susceptibility assays were performed in 96-well sterile plates, filling each well with 95 μl of YPD containing decreasing (twofold) concentrations of the antibiotic nourseothricin, ranging from 500 μg/ml to 0.78 μg/ml (including a growth control without nourseothricin for each serial dilution). Finally, each well (except for sterility controls) was inoculated with 5 μl of a cell suspension containing approximately 10^4 ^cells from an exponentially growing culture corresponding to each transformed strain under evaluation (overnight growth in SC-Ura and refreshed in YEPD). The plates were incubated in a static culture at 28°C for 40 h, and growth was determined by measuring absorbance at 550 nm in each well with a microplate reader. Each experiment was performed with at least two independent transformants.

### Western blot assays

Yeast cells were grown in YEPD overnight at 24°C to an optical density of 0.8-1 (OD_600_). The culture was then refreshed to 0.2 OD_600 _and grown at 24°C for 6 h. The procedures used for immunoblot analyses, including cell collection and lysis, collection of proteins, fractionation by SDS-polyacrylamide gel electrophoresis, and transfer to nitrocellulose membranes, have been described previously [39]. Phosphorylated Slt2/Mpk1 was detected using anti-phospho-p44/p42 MAPK (thr^202 ^/tyr^204^; Cell Signaling Technology, Beverly, MA). To monitor protein loading, actin levels were determined using mouse anti-actin mAb C4 (ICN Biomedicals, Aurora, OH). For the quantification of the bands from autoradiography films, densitometric analysis was performed using the Quantity One package (Bio-Rad Laboratories). For each sample (mutant strain), a fold-change of Phospho-Slt2 regarding the levels observed in the wild-type strain was calculated as the ratio of the intensity of the P-Slt2 band normalized by the amount of actin for each sample (mutant/wt). Experiments were carried out at least in duplicate.

### β-galactosidase reporter assays

Yeast transformants were grown overnight in an SC-Ura or SC-Ura-Leu medium, as required, at 24°C and then the culture was refreshed in YEPD to 0.2 OD_600 _and grown at 24°C for 6 h. Yeast cell extracts were prepared by harvesting cells by centrifugation from 5 ml of culture. The cells were then resuspended in 250 μl of breaking buffer (100 mM Tris-HCl pH = 8, 1 mM Dithiothreitol, 20% glycerol), and glass beads (Glasperlen ca. 1 mm, Sartorius AG, Germany) were added to break cells in a Fast-Prep machine. Finally, extracts were clarified by centrifugation and protein concentrations were measured using the Bradford method. β-galactosidase assays were performed using the crude extracts obtained as described previously [85], scaling the protocol to a 96-well microtiter plate format. 5-10 μl of cell extract was mixed with 90-95 μl of Z buffer plus β-mercaptoethanol (0.03%) and 20 μl of *o*-nitrophenyl-β-D-galactopyranoside (ONPG) (4 mg/ml in Z buffer). The absorbance of the enzymatic reaction was measured at 415 nm on a microplate reader (Model 680, Bio-Rad) after at least 10 min of incubation at 30°C and the addition of 50 μl of 1M Na_2_CO_3 _to stop the reaction. *β*-galactosidase activity was expressed as nmoles of ONPG converted/minute/mg of protein. Experiments were performed at least in triplicate involving three independent yeast transformants.

### Cell wall phenotypic analyses

Yeast cells were grown overnight at 24°C in YEPD to mid-log phase. The culture was diluted to an OD_600 _of around 0.2 and then incubated at 24°C in YEPD for 4 h. These cultures were subsequently diluted again to 0.2 (approximately 15 × 10^3 ^cells in 5 μl) and four fivefold dilution series were prepared. Finally, 5 μl of each dilution was spotted on to YEPD solid media containing 100 μg/ml Congo red (Merck), 12 mM caffeine (Sigma), 200 μg/ml SDS (Duchefa Biochemie), 50 μg/ml Hygromycin B (Roche), and 40 ng/ml caspofungin (kindly provided by Merck), using a multi-blot replicator (V&P Scientific, San Diego, CA). Growth was monitored on the plates after 2-3 days at 30°C.

### Flow cytometry and Microscopic analysis

Yeast strains were grown overnight at 24°C in YEPD to mid-log phase. The culture was diluted to an OD_600 _of around 0.2 and then incubated at 24°C in YEPD for 4 h. After this time, 1 ml of cells was collected, washed with PBS and stained with Calcofluor white (Fluorescent Brightener 28, Sigma-Aldrich, St. Louis, MO) at a final concentration of 5 μg/ml for 10 min in darkness. For the flow cytometry analysis of chitin content using Calcofluor staining, cells were analyzed with a BD LSR flow cytometer (Becton Dickinson) by acquiring fluorescence through a 380 LP filter. Cell viability was monitored by staining cells with propidium iodide (0.05 mg/ml) acquiring fluorescence through a 670 LP filter. As experimental control, stained yeasts were also analyzed by fluorescence microscopy using a Nikon TE2000 fluorescence inverted microscope equipped with a CCD camera. Digital images were acquired with an Orca C4742-95-12ER camera (Hamamatsu Photonics, Japan) and processed with Aquacosmos Imaging System software.

## Abbreviations

CWI: cell wall integrity; MAPK: mitogen activated protein kinase; GPI: glycosylphosphatidylinositol; WT: wild-type.

## Authors' contributions

PA was responsible for the assessment of levels of Slt2 phosphorylation, β-galactosidase assays, flow cytometry, phenotypic analysis and fluorescence microscopy. SD-M conducted the setting-up and subsequent screening for nourseothricin resistance with the collection of yeast mutants, and participated in fluorescence microscopy assays. RG carried out bioinformatic analysis and figure design. CN participated in the coordination of the study. JMR-P and JA conceived the study, participated in the design and analysis of experimental data and wrote the manuscript. All authors read and approved the final manuscript.

## Supplementary Material

Additional file 1 **Complete set of yeast mutants with increased levels of Slt2 phosphorylation**. Representative Western blot experiments of yeast mutants in which MAPK Slt2 was constitutively activated are shown. Click here for file

Additional file 2 **Yeast mutant strains resistant to nourseothricin without increased levels of Phospho-Slt2**. Functional information on the genes whose disruption leads to antibiotic resistance without detectable Slt2 phosphorylation is shown. Click here for file
