# Supplementary material for: Genome-wide survey of yeast mutations leading to activation of the yeast cell integrity MAPK pathway: Novel insights into diverse MAPK outcomes
Source: BMC Genomics. 2011 Aug 2;12:390. doi: 10.1186/1471-2164-12-390 (PMC3167797; doi:10.1186/1471-2164-12-390)
Supplement: Additional file 1 — Complete set of yeast mutants with increased levels of Slt2 phosphorylation. Representative Western blot experiments of yeast mutants in which MAPK Slt2 was constitutively activated are shown. [file 1471-2164-12-390-S1.PDF]

Additional file 1 (Figure S1)

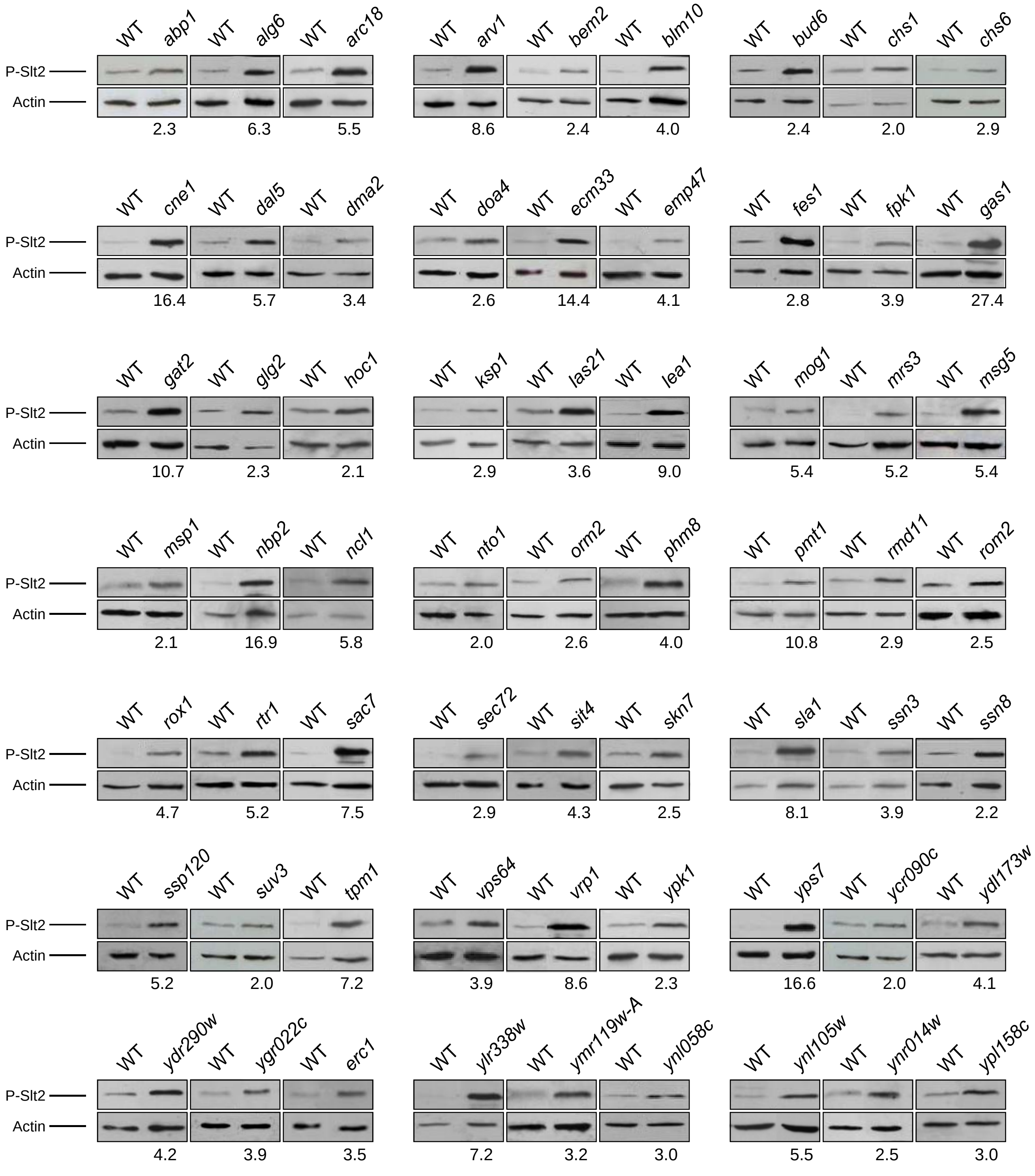

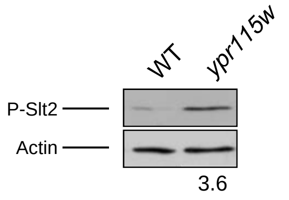

Figure S1
Complete set of yeast mutants with increased levels of Slt2 phosphorylation. Exponential phase cultures of the indicated strains were taken and processed for immunoblotting as described in “Materials and Methods”. Numbers below Western-blots correspond to the fold-change of Phospho-Slt2 in each mutant strain relative to the wild-type (WT). This value was calculated by densitometric quantification of the P-Slt2 bands normalized with respect to the actin bands, using the wild-type strain as reference (fold-change set to 1.0).
