# Supplementary material for: Genome-wide survey of yeast mutations leading to activation of the yeast cell integrity MAPK pathway: Novel insights into diverse MAPK outcomes
Source: BMC Genomics. 2011 Aug 2;12:390. doi: 10.1186/1471-2164-12-390 (PMC3167797; doi:10.1186/1471-2164-12-390)
Supplement: Additional file 2 — Yeast mutant strains resistant to nourseothricin without increased levels of Phospho-Slt2. Functional information on the genes whose disruption leads to antibiotic resistance without detectable Slt2 phosphorylation is shown. [file 1471-2164-12-390-S2.PDF]

Additional file 2 (Table S1)

S. cerevisiae mutant strains resistant to nourseothricin without increased levels of Phospho-Slt2

ORF	Gene	Functional Group	Description
YAL023C	*PMT2*	Cell Wall and Morphogenesis	Mannosyltransferase involved in O-glycosylation
YBL061C	*CHS4*	Cell Wall and Morphogenesis	Involved in the cell wall chitin biosynthetic process
YBR082C	*UBC4*	Protein Metabolism	Responsible for ubiquitin-dependent protein degradation
YBR171W	*SEC66*	Transport	Component of ER protein-translocation subcomplex
YCR081W	*SRB8*	Transcription	Component of RNA polymerase II holoenzyme and mediator (SRB)
YDR163W	*CWC15*	Nuclear	Protein possibly involved in pre-mRNA splicing
YDR241W	*BUD26*	Cell Wall and Morphogenesis	Protein that may be involved in bud site selection and bipolar budding
YDR293C	*SSD1*	Cell Wall and Morphogenesis	Suppressor of Sit4 deletion 1, regulates formation and composition of the CW
YDR315C	*IPK1*	Metabolism	Inositol polyphosphate kinase 1
YDR461W	*MFA1*	Signal Transduction	Mating pheromone a-factor
YEL048C		Unknown	Protein of unknown function
YER141W	*COX15*	Other	Protein required for cytochrome oxidase assembly
YGL007W		Unknown	Protein of unknown function
YGL151W	*NUT1*	Nuclear	Negative regulation of URS2 1. Histone acetyltransferase activity
YGL174W	*BUD13*	Cell Wall and Morphogenesis	May be involved in bipolar budding and bud site selection
YHL029C		Unknown	Protein of unknown function
YHR006W	*STP2*	Transcription	Transcription factor involved in transcription of amino acid permease genes
YHR160C	*PEX18*	Metabolism	Peroxisomal biogenesis protein (peroxin)
YIR005W	*IST3*	RNA Metabolism	Involved in pre-mRNA retention and splicing (pre-mRNA RES complex)
YJR059W	*PTK2*	Transport	Serine/threonine protein kinase required for high-affinity polyamine transport
YJR127C	*ZMS1*	Transcription	Transcription factor required for normal growth on glycerol
YML037C		Unknown	Protein of unknown function
YML109W	*ZDS2*	Other	Protein involved in regulation of transcriptional silencing and life span
YMR037C	*MSN2*	Transcription	Transcriptional activator for genes in the multistress response
YMR095C	*SNO1*	Metabolism	A glutaminase that is likely involved in pyridoxine metabolism
YMR311C	*GLC8*	Signal Transduction	Modulator of protein serine/threonine phosphatase Glc7p
YNL030W	*HHF2*	Nuclear	Chromatin binding protein that functions in chromatin related events
YNL106C	*INP52*	Stress	Inositol polyphosphate 5-phosphatase that acts in actin cytoskeleton organization
YNL127W	*FAR11*	Other	Involved in cell cycle arrest in response to pheromone
YNL236W	*SIN4*	Transcription	General RNA polymerase II transcription factor activity
YNL322C	*KRE1*	Cell Wall and Morphogenesis	Cell wall protein needed for cell wall beta-1,6-glucan assembly
YNL323W	*LEM3*	Signal Transduction	Involved in the ATP-dependent uptake of phosphatidyl-ethanolamine and -choline
YNR005C		Cell Wall and Morphogenesis	Protein that may be involved in cell wall biogenesis
YNR010W	*CSE2*	Nuclear	Component of RNA polymerase II mediator (SRB) subcomplex
YOL159C		Unknown	Protein of unknown function
YOR115C	*TRS33*	Transport	ER to Golgi vesicle-mediated transport
YPL183C		Unknown	Protein of unknown function
YPR070W	*MED1*	Transcription	A component of the RNA polymerase II holoenzyme and the mediator subcomplex

Functional categories and description were assigned based on the information provided by the BIOBASE Knowledge Library Proteome.
